# Bryophyte Spores Tolerate High Desiccation Levels and Exposure to Cryogenic Temperatures but Contain Storage Lipids and Chlorophyll: Understanding the Essential Traits Needed for the Creation of Bryophyte Spore Banks

**DOI:** 10.3390/plants11091262

**Published:** 2022-05-07

**Authors:** Giuseppe Tiloca, Giuseppe Brundu, Daniel Ballesteros

**Affiliations:** 1 Seed and Stress Biology, Royal Botanic Gardens Kew, Wakehurst Place, Ardingly RH17 6TN, West Sussex, UK; tilocagiuseppe93@gmail.com; 2 Dipartimento di Agraria, Università degli Studi di Sassari, 07100 Sassari, Sardinia, Italy; gbrundu@uniss.it; 3 Departamento de Botànica y geología, Universitat de València, 46100 Burjassot, Valencia, Spain

**Keywords:** cryopreservation, chlorophyll, desiccation tolerance, differential scanning calorimetry, ex situ conservation, in vitro germination, liquid nitrogen, lipid crystallization, unfrozen water content

## Abstract

Understanding the desiccation and freezing tolerance of bryophyte spores is vital to explain how plants conquered land and current species distribution patterns and help to develop efficient ex situ conservation methods. However, knowledge of these traits is scarce. We investigated tolerance to drying (at 15% relative humidity [RH] for two weeks) and freezing (1 h exposure to liquid nitrogen) on the spores of 12 bryophyte species (23 accessions) from the UK. The presence of storage lipids and their thermal fingerprint, and the levels of unfrozen water content, were determined by differential scanning calorimetry (DSC). The presence of chlorophyll in dry spores was detected by fluorescence microscopy. All species and accessions tested tolerated the drying and freezing levels studied. DSC suggested that 4.1–29.3% of the dry mass is storage lipids, with crystallization and melting temperatures peaking at around −30 °C. Unfrozen water content was determined <0.147 g H_2_O g^−1^ dry weight (DW). Most of the spores investigated showed the presence of chlorophyll in the cytoplasm by red autofluorescence. Bryophyte spores can be stored dry at low temperatures, such as orthodox seeds, supporting the creation of bryophyte spore banks. However, the presence of storage lipids and chlorophyll in the cytoplasm may reduce spore longevity during conventional storage at −20 °C. Alternatively, cryogenic spore storage is possible.

## 1. Introduction

The desiccation and freezing tolerance of propagules and vegetative tissues are vital traits that permitted plants to conquer land [1,2,3] and impact current plant species distribution (e.g., [4,5,6,7,8]) and can be exploited for ex situ plant conservation in germplasm banks [9,10]. Desiccation and freezing tolerance are archaic traits in the plant lineages that were likely present in the ancestors of all living things [3,11]. They evolved in ancient plants, such as green algae and the earliest land plants, producing evolutionary innovations such as cold acclimation mechanisms [1,12] or the expression of desiccation tolerance in the vegetative plant body [11,13,14,15]. Significant work has been published around the occurrence, mechanisms and evolution of the desiccation and freezing tolerance of the vegetative tissues of land plants [1,8,11,13,14,15,16]. In addition, significant work has been achieved in understanding the fundamental basis of desiccation and freezing tolerance in the reproductive structures of seeded plants (i.e., seeds and pollen) due to their crucial role in agriculture and biodiversity conservation [17,18,19,20,21,22]. However, little is known about the desiccation and freezing tolerance of spores, the reproductive structures of non-seeded land plants, across a breadth of taxa. This knowledge is not only imperative to test current hypotheses on how desiccation and freezing tolerance evolved in the plant kingdom [14] but critical to develop efficient ex situ conservation strategies for spore-bearing taxa, such as pteridophytes and bryophytes [10,23]. 

In terms of ex situ plant conservation in germplasm banks, desiccation and freezing tolerance are the traits that determine the potential long-term “storability” of the seeds, the main propagules used for the ex situ conservation of plant genetic resources [9,24]. Seeds that tolerate high desiccation levels (usually after drying at relative humidity (RH) <25% and reaching water content <0.10 g H_2_O g^−1^ dry weight (DW)) and can be stored at sub-zero temperatures (ca. −20 °C) are considered “orthodox” in relation to their storage behavior and can be stored at the conditions of “conventional” seed banks [25,26]. However, not all seeds tolerate such high levels of desiccation (so called “recalcitrant” seeds) or, if they tolerate the initial desiccation, do not tolerate the exposure to sub-zero temperatures [26]. In addition, some seeds that tolerate drying and exposure to sub-zero temperatures cannot survive for the length of time expected for long-term conservation (i.e., at least several decades to a century [27]), and some age and die faster than expected (or than desired). For example, seeds of the genera *Salix* L. and *Populus* L. are known to age and die very quickly in conventional seed banks, typically within two decades of storage, ageing even during a few decades of cryogenic storage [26,28]. Other examples of seeds that age faster than expected (if compared to the longevity found at higher temperatures such as 5 °C) when stored dry at −20°C are those of papaya, some orchids, some *Cuphea* P. Browne species and approximately 26% of the native Hawaiian flora [29,30,31,32,33]. Moreover, some temperate trees such as elm (*Ulmus glabra* Huds.) or beech (*Fagus sylvatica* L.) also have short lifespans during conventional storage [34]. The presence of active chloroplasts in the dry cells (e.g., *Salix* sp.pl. and *Populus* sp.pl.), or certain combinations of storage lipids within the dry/glassy cytoplasm that crystallize at the storage temperatures set for orthodox seed storage (e.g., orchids, *Cuphea* species), are the main issues for the short longevity of many of these seeds (reviewed in [35]). However, the exact mechanisms involved in seed deterioration in these two “dry cell architectures” are still unresolved [35]. 

In this context, the spores of lower plants have been highlighted as unique unicellular models to study stress tolerance [36], which could also provide insights into potential mechanisms that influence seed lifespan during storage [37]. While fern spores have been used in previous research [35,36,37], in this paper, we have approached the bryophyte spores. We aimed to (1) provide a novel unicellular model to study storability and longevity issues that occur in seeds and other dry germplasm, and (2) reinforce the knowledge of bryophyte spore traits that are vital for the correct development of bryophyte spore banks. Bryophytes, the second largest group of land plants, comprise approximately 20,000 species occupying diverse habitats on all continents, from the tropics to polar regions [38]. While bryophyte desiccation tolerance in the gametophyte has been relatively well studied, it has been indicated that more research is needed in relation to the spore [14]. Survival of the bryophyte’s spores under desiccation and freezing exposure has been qualitatively well described (e.g., [39]), but more data quantifying these responses are still needed. Moreover, it is important to investigate which traits affect spore storability, such as chloroplast and storage lipid presence [35], as well as the thermal fingerprint of these lipids (e.g., [40,41]). Finally, it is important to consider the occurrence of these traits with a phylogenetic perspective and to investigate their relation to other plant groups, such as pteridophytes and seeded plants. 

For this research, we have empirically measured and quantified the desiccation and freezing tolerance, as well as other traits involved in the potential storability, of bryophyte spores from diverse temperate species. We have used a multidisciplinary cryobiotechnological approach that involves biophysics, ecophysiology, cytology and in vitro technology, applying innovative and non-invasive techniques developed for seed science to bryophyte spores. The hypotheses to test were as follows: (1) bryophyte spores from a variety of species tolerate high desiccation (to 15% RH), (2) dry bryophyte spores from a variety of species tolerate exposure to sub-zero temperatures (liquid nitrogen (LN) exposure), and (3) dry bryophyte spores tolerate freezing because there is no “freezable water” in their cells when dried. We also expected that, analogously to seeds and fern spores [35], dry bryophyte spores containing chlorophyll (in chloroplasts) show low storage lipids (<10%), whereas relatively high storage lipids (>10%) are found in non-chlorophyllous spores. The presence of these two traits, as reasoned above, is important to determine potential long-term storage issues in bryophyte spores. The increasing threats to bryophyte diversity and the global lack of ex situ conservation programs demand the urgent exploration of traits that are vital for the establishment of new biobanking initiatives [42,43,44]. In this respect, this paper also aims to support the preservation of spore-bearing plants by providing empirical data to enable the formation of bryophyte spore banks. This ex situ conservation approach has been previously recommended [10,23,43] but is not in wide use (to our knowledge, there are no bryophyte spore banks in the world). We believe that spore storage can be of high value to support IUCN conservation missions for bryophytes and will complement and strengthen previous ex situ conservation initiatives for bryophyte biobanking, such as, for example, the in vitro collections and cryobanks for bryophyte gametophytes established in the late 1990s and early 2000s at the Royal Botanic Gardens Kew [42], the Cincinnati Zoo and Botanical Garden [45] and the Bryophyte Biology Group Belgrade at the University of Belgrade [43]. 

## 2. Results

### 2.1. Germination of Bryophyte Spores after Desiccation and Freezing 

Spores from all bryophyte species (12) and accessions (23) tested were germinated on Knop medium after being harvested and dried to 15% RH for 2 weeks (Table 1), indicating a high degree of desiccation tolerance in the spores of all species tested. Spore water content (WC) ranged between 0.005 and 0.066 g H_2_O g^−1^ DW as measured in the four species scanned by Differential Scanning Calorimetry (DSC), for which enough spores were available for WC determinations. From the 23 accessions (acc.) tested, 16 (70% of the total) reached 100% germination and seven accessions reached less than 100% germination, four of which (17% of the total accessions) had germination <50%. For most species tested, germination was consistent among accessions. For example, *Bryum capillare* Hedw. and *Polytrichum formosum* (Hedw.) G.L. Sm. showed 100% germination in all accessions tested and *Funaria hygrometrica* Hedw. showed 100% germination in two accessions and near 75% in the third accession tested. Germination was largely different between accessions only for *Kindbergia praelonga* (Hedw.) Ochyra (100% in acc. 1 and 21% in acc. 2). First germinated spores were detected four days (96 h) after sowing in all the species tested, except for a *B. capillare* (acc. 3) and *Ceratodon purpureus* (Hedw.) Brid. for which germination started one day (24 h) after sowing, and for *Orthodontium lineare* Schwägr. (acc. 2) for which germination started eight days (192 h) after sowing. These observations were supported by the time to reach 50% of the final germination percentage (t_50_) (Table 1), which was generally very short (≤3 days) in most *B. capillare* accessions, *C. purpureus* and two *F. hygrometrica* accessions, and very large (>10 days in *O. lineare* (acc. 2), whereas most species/accessions ranged between 3 and 6 days.

After exposure to LN temperatures (−196 °C), all species and accessions showed some degree of germination, except for one of the accessions of *K. praelonga* (acc. 2) and *Marchantia polymorpha* L. for which spores did not germinate. From the 23 accessions tested, 11 (48% of all accessions tested) reached 100% germination regardless of LN exposure, five accessions showed a significant decrease (*p* < 0.05, prop.test) in germination from 100% to <90% following exposure to LN, and seven accessions reached less than 100% germination in both treatments. In this last group, only three accessions showed a significant decrease in germination (*p* < 0.05, prop.test) following LN exposure (Table 1). In summary, 65% of the accessions tested showed no significant decrease in germination after LN exposure and 35% of accessions showed significantly lower germination after LN exposure. All the t_50_ measurements showed similar results before and after LN exposure, except for 35% of the accessions tested that showed significantly larger t_50_ in LN-exposed spores (*Atrichum undulatum* (Hedw.) P. Beauv., *B. capillare* (acc. 1), *Dicranum scoparium* Hedw., *F. hygrometrica* (acc. 1 and 3), *K. praelonga*, *Leptobryum pyriforme* (Hedw.) Wilson and *O. lineare* (acc. 1)) or *O. lineare* acc. 2, which showed significantly shorter t_50_ in LN-exposed spores. These results support a high degree of freezing tolerance in the spores of most bryophyte species and accessions tested.

### 2.2. Calorimetric Properties of Bryophyte Spores in Relation to Storability by Differential Scanning Calorimetry (DSC)

**Figure 1:**
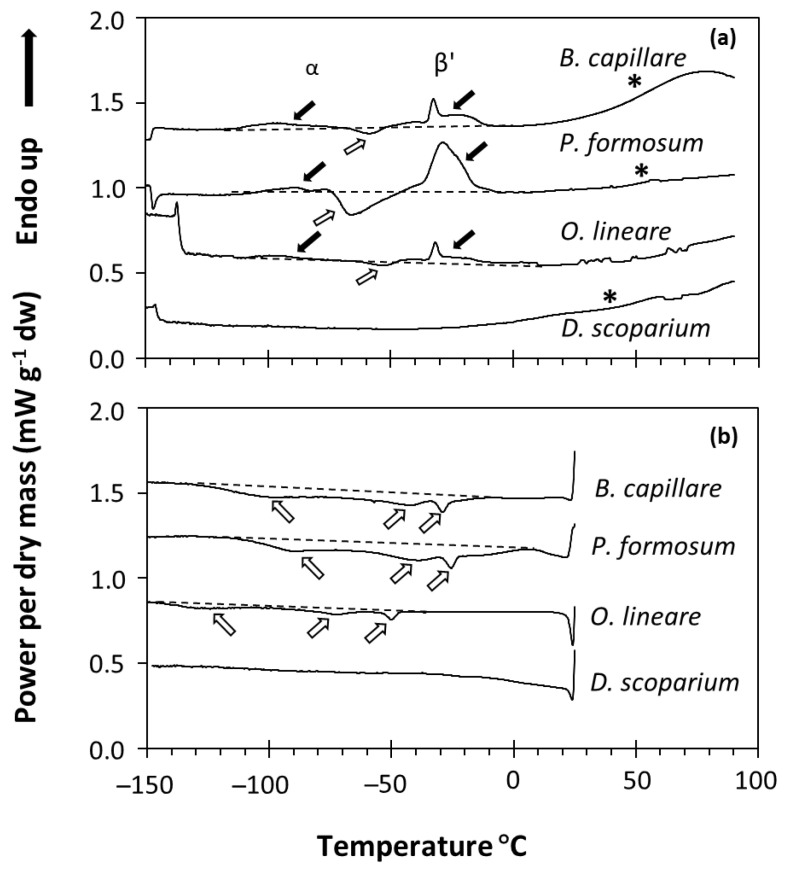
DSC melting (**a**) and cooling (**b**) scans of four bryophyte species (*Bryum capillare* acc. 2, *Polytrichum formosum* acc. 2, *Orthodontium lineare* acc. 3, *Dicranum scoparium* acc. 4). Black arrows indicate lipid melting transitions, usually occurring with two events in the melting scans (**a**): one around −80 or −90 °C (related to α crystals [46]) and one around −30 °C (related to β’ crystals [46]). White arrows indicate lipid crystallization transitions, usually occurring with three broad events around −90, −50 and −25 °C in the cooling scans (**b**), but also as a lipid recrystallization event around −60 or −70 °C in the warming scans (**a**). A dashed line was added to indicate the baseline of the scan considered. Asterisks indicate the broad second-order transition that marks the glass transition.

**Figure 2:**
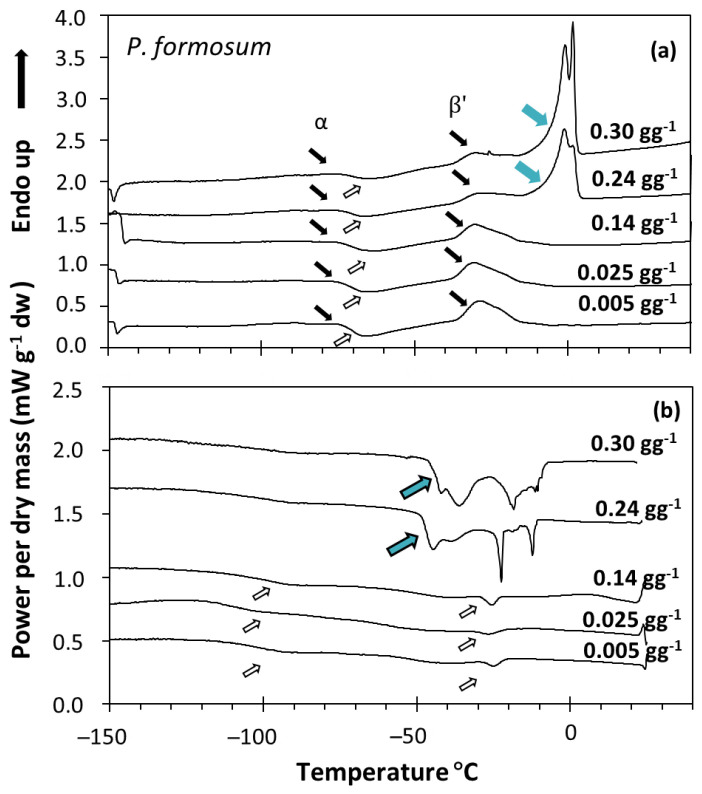
DSC melting (**a**) and cooling (**b**) scans of *Polytrichum formosum* acc. 1 spores containing the indicated water content. Samples were scanned at 10 °C min^−1^ from 25 to −150 °C and then from −150 to 40 °C. Lipid melting transitions are expressed using black arrows for the melting events and white arrows for the crystallization events (as in Figure 1). When water content increased, other peaks appeared (blue arrows), increasing the enthalpy of the main event (∆Hm) and occurring at 0 °C in the melting scans (**a**). These peaks were attributed to water melting (**a**), and water + lipid crystallization (**b**).

The physical state of water and lipids in the spores of four bryophyte species was studied using DSC. Only four of the species harvested were used due to the small amounts of bryophyte spores collected and the sample size needs of the DSC technique (see Materials and Methods). DSC determines changes in the heat capacity of a sample over a temperature scan, so the temperature (°C) in which crystallization (exothermic events) and melting (endothermic events) transitions occur and the enthalpy associated with these events can be measured (Figure 1). Dry bryophyte spores (equilibrated at RH ≤ 75% and containing 0.012 to 0.16 g H_2_O g^−1^ DW) showed different phase changes (first-order transitions) between −120 and 0 °C in both the melting (Figure 1a) and cooling (Figure 1b) scans. As this signal did not change with the water content of the sample (see, e.g., *P. formosum* samples in Figure 2), we determined that it corresponded to the melting and crystallization of the spore storage lipids (mainly triacyclglycerols (TAG)). Lipid melting transitions were relatively complex, with diverse exothermic peaks in the cooling scans (Figure 1b) and a combination of endothermic and exothermic peaks in the melting scans (Figure 1a). This complex behavior can be attributed to the polymorphism of the TAG during their crystallization and melting. For example, TAG crystallized during the cooling scans at a kinetics determined by the cooling rate (10 °C min^−1^). During the melting scan, the melting profile of these crystallized TAG included different endothermic and exothermic peaks related to the different crystal forms [46]. These were attributed to the melting of the α crystals (broad endothermic peak between −120 and −70 °C), followed by a re-crystallization event of these melted α crystals (exothermic peak between −70 and −50 °C), finally followed (large endothermic peak between −50 and 0 °C) by the melting of the β’ crystals [46]. Only the *D. scoparium* scan showed no transitions in both the cooling and melting scans (Figure 1a,b). In addition to the first-order transitions, some scans also showed a broad second-order transition (i.e., wide step of the heat flow at around 60 °C indicated by an asterisk in Figure 1), which was very apparent in *B. capillare* and *P. formosum* spores. These second-order transitions have been related to a glass transition in pollen and fern spores [17,37,47] and suggest that the bryophyte spores tested were in the glassy state at room temperature after drying to 15% RH.

**Figure 3:**
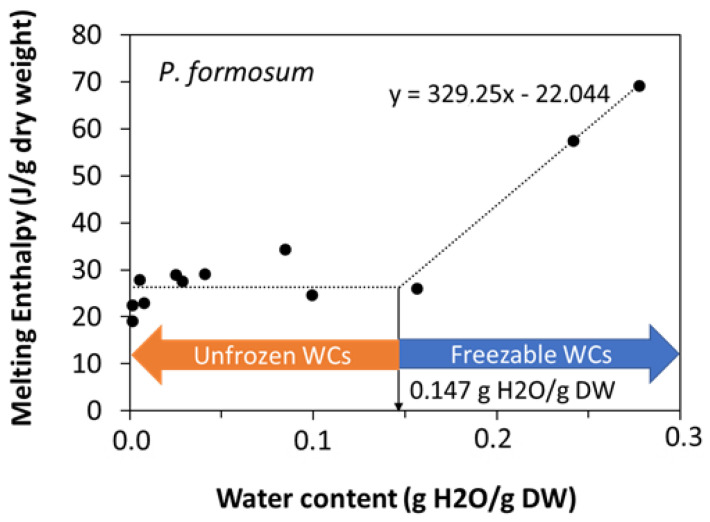
Relationship between water content (g H_2_O g^−1^ DW) and enthalpy of melting transition (J g^−1^ DW) measured for spores of 12 samples of *Polytrichum formosum* (acc. 1 and acc. 2) with diverse water content. The slope of the linear relationship of the two wettest samples (see equation in the figure panel) was used to calculate the ΔH of the water melting or freezing transition on a g^−1^ H_2_O basis. The intersection between this line and the horizontal line attributed to the TAG transitions shows the exact point below which melting or freezing transition cannot be observed [40].

When the water content increased above a certain threshold, other peaks appeared in the scans, which increased the enthalpy of the main melting event (ΔHm) as a function of the water content of the sample, and moved towards 0 °C. These events were related to ice melting (Figure 2a) or water crystallization (Figure 2b), as observed in other spores and seeds (e.g., [40]). The effect of water content on crystallization and melting enthalpy can be visualized in the spores of *P. formosum* from plots of enthalpy (Figure 3), calculated on a g^−1^ dry weight basis, versus water content, expressed on a g^−1^ dry weight basis (melting transitions given in Figure 1 and Figure 2). At low water content, when only the lipid transitions were detected, ΔHm was constant with water content and averaged 25.8 J g^−1^ DW (Table 2, Figure 3). Once spores were sufficiently hydrated, ∆Hm increased linearly with increasing water content. The value for ∆H for the water melt (∆HH_2_O, slope of linear regression) was 329 J g^−1^ H_2_O (Table 2, Figure 3). The amount of water that did not freeze was calculated from the intersection of a horizontal line drawn for lipid transitions and the sloped line drawn for water transitions (Figure 3). Unfrozen water content was below 0.147 g H_2_O g^−1^ DW (Table 2, Figure 3), indicating the point at which spores of *P. formosum* can be stored without formation of lethal ice crystals.

Finally, the size and temperature of the TAG melting transitions allowed us to predict the predominant fatty acid of the TAG and estimate the lipid content for the diverse species (after [40]). Based on the melting temperatures, the TAG in the spores were predicted to contain mostly linolenic acid (Table 2). Lipid contents were estimated between 4.1 and 29.3% for the spores of *O. lineare* and *P. formosum*, respectively (Table 2).

### 2.3. Chlorophyll and Chloroplast Presence in Spores by Fluorescence and Optical Microscopy 

**Figure 4:**
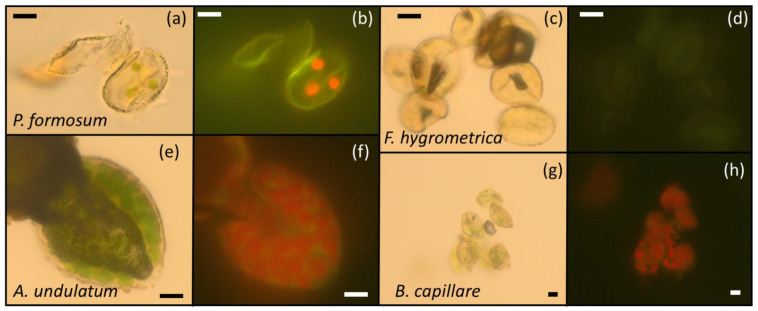
Microscopy images of the spores of *P. formosum* (**a**,**b**), *F. hygrometrica* (**c**,**d**), *A. undulatum* (**e**,**f**) and *B. capillare* (**g**,**h**). *(***a**,**c**,**e**,**g**) were taken by an optical microscope under white light. (**b**,**d**,**f**,**h**) were taken by epifluorescence after excitation at 450–480 nm (blue filter). Green plastids or chloroplasts are clearly visible in *P. formosum* (**a**), *A. undulatum* (**e**) and *B. capillare* (**g**), particularly due to the red autofluorescence emitted (**b**,**f**,**h**), respectively.

Spores collected from 36 of the accessions harvested, corresponding to 21 different species (Table 3), were observed under the microscope to visualize the presence of green plastids or chloroplasts (optical microscopy, Figure 4a,e,g) or red autofluorescence of the chlorophylls (fluorescence microscopy, Figure 4b,f,h). The rest of the accessions harvested (Table 3) did not have spores or all spores were used in the germination experiments. Under white light, spores containing green plastids or chloroplasts were seen in 29 (80%) of the samples tested, corresponding to 20 (95%) of the species tested (Table 3). With epifluorescence microscopy, red autofluorescence in the spores was detected in 30 (83%) of the samples tested, corresponding also to 20 (95%) of the species tested (Table 3). Observations made on the presence of green plastids and red autofluorescence were similar among accessions of the same species (e.g., *Dicranella heteromalla* (Hedw.) Schimp., *Hipnum cupressiforme* Hedw., *K. praelonga*, *Orthotrichum affine* Schrader ex Bridel, *Tortula muralis* Hedw.); however, there were some species for which some variation in the observations was found (Table 3). For example, accessions 1–4 of *B. capillare* showed the presence of green plastids and red autofluorescence, while none of these were detected in accession 5. Similarly, accessions 1, 3 and 7 of *F. hygrometrica* did not show the presence of green plastids and red autofluorescence (Figure 4c,d), while these were detected in accession 8. We do not have any explanation for this variation. We speculate that it could have been related to the spore maturity stage in *F. hygrometrica* (immature spores could have shown the presence of red autofluorescence) or spore age in *B. capillare* (aged spores could have shown a lack of red autofluorescence).

## 3. Discussion

The results of this study demonstrate that bryophyte spores from a diversity of species (12) growing in a temperate environment (UK) are highly desiccation- and freezing-tolerant, surviving, in all cases, two weeks of drying at 15% RH (to WCs ranging from 0.005 to 0.066 g H_2_O g^−1^ DW) and, in most cases, a brief (1 h) exposition to LN temperatures. Tolerance of the dry spores to sub-zero temperatures was likely possible due to the removal of the fraction of intracellular water that can freeze (<0.147 g H_2_O g^−1^ DW) and the vitrification (i.e., glass formation) of the cytoplasm upon drying. These results, in combination with previous research on the desiccation and freezing tolerance of bryophyte spores (e.g., [39,50,51,52,53]), indicate that spores can be stored dry at low temperatures, supporting the creation of bryophyte spore banks for ex situ conservation purposes [10,23]. However, this study also showed that most spores tested presented in the cytoplasm storage lipids (TAG) with crystallization and melting signals between 0 and −120 °C that peaked at around −30 °C, which appeared always in combination with green plastids loaded with chlorophyll. These two traits alone may influence (i.e., reduce) spore longevity during conventional storage at −20 °C, as in the case of oily or chlorophyllous seeds and fern spores [35]. This potential reduction in spore longevity could be more relevant in species such as *P. formosum*, which showed an interesting and unique combination of high TAG content (near 30%) and the presence of chloroplasts. This combination has not been previously observed in seeds and fern spores [35]. As an alternative for the long-term conservation of these propagules, cryogenic spore storage is suggested.

### 3.1. Tolerance to Desiccation of Bryophyte Spores

The desiccation tolerance of bryophyte spores is widely recognized [11,13,14,15] and generally known from reports of bryophyte spore longevity in the dry state (achieved at RHs between 40 and 75%). Data were often obtained from spores preserved for several years in herbaria sheets stored under the standard ambient conditions of an herbarium (e.g., [54,55]). However, also, data came from some drying experiments designed to understand the long-distance dispersal of bryophyte spores [50,51] or to prepare spores for dry cryogenic storage [52,53]. Our results indicating high viability (100%) in the majority (70%) of species tested after drying at 15% RH and 15 °C for 2 weeks to WCs < 0.07 g H_2_O g^−1^ DW confirm these previous observations. The lower viability (50–100% or <50%) in the rest of the species tested after drying (30% of the total) suggests different possibilities: (1) spore recalcitrance (i.e., sensitivity to desiccation, particularly in *M. polymorpha* and *A. undulatum*, which showed 7% and 25% germination after drying, respectively); (2) short spore longevity in the dry state (i.e., relatively fast ageing during the duration of the drying (2 weeks at 15% RH) and the time for which they were stored dry at 4 °C until the beginning of the germination experiments (up to 4 weeks, see Materials and Methods)); (3) the presence of some form of dormancy that was not broken during the in vitro germination in the culture medium and (4) the low initial quality of the spores harvested. Our data do not support that the spores used in these experiments were recalcitrant (desiccation-sensitive). For example, recalcitrant seeds (or pollen or spores) are those that do not tolerate drying <50% RH or when water content decreases <0.10 g water/g dry weight [18,26,36,47]. In other words, they fully die when dried to such low moisture content. All accessions tested in this paper tolerated, to some extent, desiccation at 15% RH for at least 2 weeks, reaching water content <0.08 g water g^−1^ DW, plus several weeks of dry storage at such conditions. Hence, we cannot consider, analogously to seeds, that they are “recalcitrant”. This is also supported by observations made by other authors, who found that some of the species with low germination after drying in our experiment are indeed highly tolerant to drying (e.g., *M. polymorpha;* [51]). In addition, our results show that the low germination after drying in one accession of *K. praelonga* was not shared by the other accession tested (which showed 100% germination after drying; see Table 1), suggesting that the spores of this species are highly tolerant to the “intense” drying treatment investigated. As indicated above, the low initial germination percentages after drying could have been promoted by the relatively fast ageing of the spores that occurred from harvest to germination (which represents up to 6 weeks of “storage” time), the presence (or induction after dry ripening) of dormancy or simply the low initial quality of the spores harvested. However, we cannot confirm any of these three hypotheses due to the lack of control germination data on freshly harvested spores, something that deserves further study in future experiments.

These results of the desiccation tolerance of bryophyte spores based on survival under the stress induced by the removal of water are also supported from a physicochemical point of view. For example, bryophyte spores dried at room temperature to low RH (15–50%) not only survive drying but also present a vitrified cytoplasm (i.e., the aqueous phase of the cytoplasm appears as a non-crystalline amorphous solid known as glass). This has been measured for *P. formosum* by Fourier transform infrared spectroscopy (FT-IR) [56] and detected in our DSC melting scans by broad second-order transitions (Figure 1). The survival to the formation of intracellular glasses is characteristic of desiccation-tolerant seeds, pollen, fern spores and many other anhydrobiotes [18,35,56,57,58,59,60], and has been suggested as an objective measure to account for desiccation tolerance in plant propagules [26,47]. 

Finally, from an ecological point of view, it is important to highlight that the spores used in this experiment were from species of temperate origin, where dispersal usually occurs under dry conditions (xerochastic dispersal) [61] and, once spores are released, they must possess desiccation tolerance as effective agents of dispersal [11]. It would be interesting to check if the desiccation tolerance observed also occurs in spores released in wet and humid conditions (hygrochastic dispersal), rarely detected in some temperate species [61] but which may be more common in tropical species. In this sense, diverse tropical species, mainly from the understory of wet primary rainforests, appear to have short longevity in the dry state or even limited tolerance to drying (at 40–50% RH, [51]). Spore collection from tropical areas of Madagascar for desiccation and freezing experiments is ongoing as a second phase of this research.

### 3.2. Tolerance of Bryophyte Spores to Sub-Zero Temperatures 

The high tolerance of bryophyte spores to freezing temperatures (around −30 °C), was extensively described by Bernard Otto van Zanten in his experiments on the long-distance dispersal of mosses and liverworts from the southern hemisphere (e.g., [39,50,51]. More recently, Qiu-Ping and Li-Wei [52,53] have determined the high capacity of spores to germinate after exposure and storage at sub-zero temperatures by testing spore germination in seven Chinese species of mosses after drying and exposure to −20 °C and storage at LN temperatures (−196 °C) for between 1 and 180 days. Our results on the germination of dry spores in a variety of UK bryophytes after LN exposure confirm these previous observations and reinforce the high tolerance of bryophyte spores from both hemispheres to sub-zero temperatures. Significant decreases in germination percentage between dry spores before and after LN exposure were shown on only 35% of the species tested. We attribute this germination reduction to the low initial germination of some of the dry spores used (e.g., *M. polymorpha, A. undulatum* and acc. 3 of *K. praelonga* had germination < 25%). However, it would be interesting to check in future experiments if there was some interaction between the intense drying treatment used (15% RH) and the exposure to the ultra-low temperatures of LN. For example, damage due to over-drying has been suggested for seeds dried to very low moisture conditions and stored at cryogenic temperatures [62].

The tolerance of the dry spores to sub-zero temperatures (Table 1) is also supported from a physicochemical point of view. For example, we have roughly determined in *P. formosum* spores (the only species for which we harvested sufficient quantities to run several DSC scans at different moisture conditions) that the water content level at which ice melting events cannot be detected at the cooling and warming rates used was 0.147 g H_2_O g^−1^ DW. Our dry spores had water content far below this limit (0.005 to 0.066 g H_2_O g^−1^ DW). This indicates that it is likely that the fraction of intracellular water in the dry bryophyte spores tested did not have the freedom to aggregate, nucleate into ice crystals and grow, and may be strongly associated with the macromolecules forming the amorphous solid (glass) at which the cytoplasm of the dry spores was found (see previous subsection). The levels of unfrozen water content measured were relatively low compared to those found in multicellular systems such as seeds and seed embryos (typically between 0.25 and 0.30 g H_2_O ^−1^ DW) but appear in the range of other unicellular systems such as fern spores [37,40]. The enthalpy of melting transitions may also reveal the properties of water and ice relevant for preservation at sub-zero temperatures. For example, pure water melts with an enthalpy of 333 J g^−1^ H_2_O, and in plant systems that tolerate drying and freezing, it is expected that lower enthalpies reflect the presence of solutes or matrices that limit ice formation or promote recrystallization [40]. We determined an enthalpy of 329 J g^−1^ H_2_O, very close to that of pure water and higher than detected in fern spores (around 230 J g^−1^ H_2_O) or other dry systems (around 150 J g^−1^ H_2_O) [40]. The high values for melting enthalpy suggest very limited endogenous protection from ice crystallization and growth during exposure to sub-zero temperatures when bryophyte spores are hydrated to WC > 0.147 g H_2_O g^−1^ DW [40]. However, this level of hydration is not needed to safely store bryophyte spores at sub-zero temperatures (based on our LN exposition results and previous research). In addition, we may need further investigations into this subject as we only performed these calculations in one species, and the determination of the unfrozen water limit was based on only two data points at the high moisture end of the analysis. 

### 3.3. Calorimetric Properties of Storage Lipids, Chlorophyll Presence and Bryophyte Storability 

This study has shown that the spores of four out of five bryophyte species tested presented storage lipids (TAG) in the cytoplasm with crystallization and melting signals between 0 and −120 °C. Based on the temperature and enthalpy of melting of the main peak detected in our DSC scans (Figure 2), TAG contents were estimated to range between 4.1 and 29.3% of the dry mass of the spore and appeared to be formed mainly of linolenic acid (Table 2). Linolenic acid is an unsaturated fatty acid typically found in fern spores [40] and seeds of temperate origin [63] and also seems to be the main fatty acid in the spores of the UK bryophyte species studied. The fatty acid composition of the storage lipids seems to be related to the ability of the propagule to cope and germinate at the typically lower temperatures of temperate habitats [63]. In this sense, it would be interesting to investigate whether the fatty acid composition of bryophyte spores changes based on their biogeography, i.e., if tropical species present a higher abundance of saturated fatty acids. From the point of view of the storage stability of bryophyte spores in ex situ conservation collections, it has been indicated that the presence of TAG in seeds and fern spores that crystallize and melt at the standard temperatures used for storage (around −20 °C) may affect (i.e., reduce) their longevity (reviewed in [35]). The TAG detected in the bryophyte spores studied showed large crystallization and melting peaks at around −20 and −30 °C, respectively (Figure 2), suggesting potential longevity issues during storage at −20 °C. However, the sole presence of storage lipids is not synonymous with longevity issues at −20 °C [35,41,64,65]. Indeed, in most cases, these issues are related to monosaturated and saturated fatty acids that compose TAG with high melting temperature ends or TAG with many melting and recrystallization events found around −20 °C (e.g., [31,41]). This question opens two areas of research in bryophyte spores: (1) to investigate whether bryophyte spores from tropical origins accumulate more saturated fatty acids in their cytoplasm with higher melting temperatures, and (2) to investigate potential longevity issues of bryophyte spores during long-term low-temperature dry storage.

Interestingly, these relatively large amounts of storage lipids appeared in combination with the presence of green plastids loaded with chlorophyll in some species (e.g., *B. capillare* and *P. formosum*). Chloroplast/chlorophyll presence in dry seeds and spores has also been related to a reduction in longevity during storage through oxidative mechanisms induced by the activity of the photosystems in the dry state, but, generally, these propagules are not loaded with many storage oils [35]. The combination of high lipid content and the presence of chloroplasts/chlorophyll brings up a particular type of “dry architecture” (as per [35]) that seems unique in bryophyte spores and that deserves further study in terms of storage stability and mechanisms of ageing in the dry state.

### 3.4. Practical Considerations from the Results to Improve the Ex Situ Conservation of Bryophytes

Bryophyte conservation efforts have recently accelerated, especially after an increase in the number of species included in the IUCN Red List of threatened species. For example, in the European continent, 22.5% of the assessed species are endangered (i.e., at high risk of extinction) [66]. Bryophyte conservation is usually performed via living collections in botanical gardens or other botanical institutions, by in vitro collections or through cryopreservation [10,23,42,43,44,67]. These collections usually preserve living tissues as gametophytes due to their year-round availability in the field, securing the future reintroduction of threatened species into the environment [23]. 

The high desiccation and freezing tolerance of bryophyte spores indicates that these propagules could also be potentially stored in ex situ conservation collections [10,23,43], allowing for the preservation of a large genetic diversity in a very reduced space, as indicated for fern spores [68]. However, while storing fern spores has increasingly become a standard method for the long-term ex situ preservation of this group of plants [68,69,70], this approach has not been extended to the bryophytes. Our results show that dry bryophyte spore storage at sub-zero temperatures is possible, and bryophyte spore banks could likely be placed at the same conditions of conventional seed banks (drying to 15–25% RH and storing near −20 °C, [25]). However, as discussed in the above sections, we cannot rule out the possibility of longevity issues in some spores due to the crystallization and melting kinetics of the spore storage lipids and the presence of plastids with chlorophyll [35]. In this case, cryogenic storage of bryophyte spores dried <0.147 g H_2_O g^−1^ DW appears as an alternative (or a back-up solution) for the long-term conservation of these propagules. In this respect, we have shown the high tolerance of the dry spores of many temperate species to LN exposure. Additionally, the safe LN storage of dry bryophyte spores for up to 180 days has been demonstrated [52,53], including some taxa present in our work (*F. higrometrica*, *Dicranum* sp.pl., *Bryum* sp.pl.).

## 4. Materials and Methods

### 4.1. Plant Material and Spore Desiccation Treatment

Bryophyte samples were collected in different locations and from varying types of habitats, and each collection was considered a different accession (Table 3). We focused on the sporophytes, as they are the structures containing the spores. Sporophytes were collected during the summer months of June and July 2021 in the wild and semiwild areas around Wakehurst Place, Royal Botanic Gardens Kew (Selsfield Rd, Ardingly, RH17 6TN; see Table 3 for details). Sporophytes were collected integrally with their seta when mature, following the methods explained in [38], using sterilized tweezers and Eppendorf tubes. At least 5 sporophytes per plant were collected. 

Open Eppendorf tubes with sporophytes were placed in a drying room (set at 15 °C and 15% RH) for two weeks to allow sporophytes and spores within them to dry. At the end of this drying treatment, Eppendorf tubes were closed, and samples were stored in a fridge (4 °C) until the beginning of the experiments (within 2 months of harvest). To conduct the germination and calorimetry experiments (see below), the sporophytes were slightly crushed with the tip of a sterilized tweezer inside the Eppendorf tubes and then homogenized with a vibrator. This process allowed the release of the dry spores in the Eppendorf tubes, which was monitored using a microscope. Only those samples containing a visible quantity of spores, or more than five mature sporophytes, were chosen for the germination and calorimetry experiments.

Other plant tissues (gametophytes) were also collected for identification using sterilized tweezers and collecting paper packets, placed in the drying room for two weeks, at the end of which they were sent to the Natural History Museum (NHM), London, for identification using the taxonomical expertise kindly provided by Dr. Silvia Pressel. The 68 accessions collected corresponded to 25 confirmed species (Table 3). 

### 4.2. Germination Tests for Desiccation and Freezing Tolerance Experiments

Germination tests were performed in 12 of the species (23 accessions) collected (*Atrichum undulatum, Bryum capillare, Ceratodon purpureus, Dicranum scoparium*, *Funaria hygrometrica, Kindbergia praelonga, Leptobryum pyriforme, Marchantia polymorpha*, *Mnium hornum Hedw., Orthodontium lineare, Polytrichum formosum* and *Tortula muralis*), as they were the only species/accessions that had clearly released spores from the sporophytes. Two conditions were tested for each species/accession: (1) spore germination after initial desiccation (15% RH) and without liquid nitrogen (LN) exposure and (2) spore germination after desiccation and 1 h of exposure to LN.

Knop medium was used as the culture medium for the bryophyte spore germination tests, following the recipe of Reski and Abel [71]. In detail, four stock solutions were prepared containing: 25 g/L KH_2_PO_4_, 25 g/L KCl, 25 g/L MgSO_4_ and 100 g/L Ca(NO_3_)_2_. To produce 1 L of Knop medium, 10 mL of each stock solution was taken; then, 0.3 g FeSO_4_ was added, and this was finally topped up with distilled water. To solidify the culture medium, it was added to 1.2% (*w*/*v*) agar (12 g/L). The medium was sterilized by autoclaving at 120 °C and 1 atmosphere for 25 min. After sterilization, and when the medium was still liquid, approximately 10 mL of the Knop medium was poured into 6-mm-diameter Petri dishes for its solidification. Plates were stored in the fridge until use. 

For the germination tests, the spores extracted (as explained above) were divided into two tubes, a 1.5 mL Eppendorf tube (for the samples before LN exposure) and a 2 mL cryovial (which was used for the LN exposition treatment). Spores without LN exposition were sown onto Petri dishes containing the Knop medium by adding 1 mL of distilled water inside the Eppendorf tubes using a pipette, suspending the spores, pouring 500 mL of this suspension into two plates and spreading the liquid over the medium. For the LN treatment, spores were exposed to LN for 1 h and then thawed on the lab bench at room temperature (around 21 °C) for 15 min, and finally sown onto Petri dishes using the same procedure indicated above. All the procedures described were conducted under a laminar flow hood to avoid cross-contamination with other species. Petri dishes containing the spores sown on medium were placed in an incubator (20 °C, 16/8 h photoperiod) and checked for germination.

Germination was scored using a light microscope the day after sowing and every 3-4 days until germination was steady or it was impossible to determine if new spores were germinating (due to large protonema growth). Germination was measured selecting a random area of the Petri dish and determining the number of spores germinated and ungerminated, and the proportion of germinated spores per treatment was calculated. This random counting was performed on at least 200 spores per Petri dish. 

In order to compare whether the germination of the spores was significantly different before and after exposition to LN, we ran a test of equal proportions for each species and accession. After checking that the proportion of germination between dishes was similar, we pooled the spore germination counts from both Petri dishes used for the germination tests. Proportions were considered to follow a binomial distribution, so a test of equal or given proportion was carried out to quantify whether the proportions (probabilities of success) in a group were the same, or whether they were equal to certain given values (prop.test, R Core Team, 2021). The significance level was taken as 0.05 so that the null hypothesis of no difference was rejected if *p* < 0.05.

### 4.3. Calorimetric Analysis of Bryophyte Spores 

The calorimetric analysis of the bryophyte spores was conducted by differential scanning calorimetry (DSC) using a Mettler Toledo DSC-1 (Mettler-Toledo AG, Analytical CH-8603 Schwerzenbach, Switzerland), calibrated for temperature with indium (156.6 °C) standards and for energy with indium (28.54 J g^−1^). DSC determines changes in the heat capacity of a sample when cooled and/or warmed and is very useful to detect crystallization and melting transitions (e.g., in water solutions and lipids), including those occurring in biological cells and tissues [17,31,40,72]. This type of experiment requires a relatively large amount of material (ideally 1 mg), which is easy to obtain when working with large samples (e.g., seeds, [31,72]) or with small samples that are produced in high quantities (e.g., fern spores, pollen [17,40]). However, due to the small amounts of bryophyte spores in the samples collected, the DSC experiments were only performed on four of the species harvested (*B. capillare*, *D. scoparium*, *O. lineare*, *P. formosum*), for which at least 0.2 mg of spores were collected. To investigate the lipid and water crystallization and melting properties of the spores, a total of 20 scans were run: 2 for *B. capillare*, 1 for *D. scoparium*, 3 for *O. lineare* and 14 for *P. formosum.*

Spores were obtained from the dry capsules stored at 4 °C (see ‘Plant Materials’), following the same procedure as the one used for germination tests. Between 0.2 and 2.2 mg of spores per species were placed inside a DSC pan, which was subsequently sealed with a lid and weighed to determine the fresh weight of the spore sample. Weights were taken using a Mettler Toledo XPR Microbalance (Mettler-Toledo GmbH Laboratory Weighing, 8606 Greifensee, Switzerland). DSC experiments were mostly conducted at a single moisture content, which was that reached after the spores were initially dried at 15% RH (see ‘Plant Materials’). Only *P. formosum* spores were produced in sufficient quantities to allow multiple DSC scans with samples at different moisture content. For these spores, moisture content was manipulated in hermetic chambers using saturated salt solutions of LiCl (which provided 11% relative humidity (RH)), NaCl (75% RH), KCl (81% RH) and KNO_3_ (94% RH) or distilled water (100% RH) for 2, 6, 8, 20, 26 h. Spore moisture content was manipulated in spores sitting in an open DSC pan, which was sealed with its lid after the moisture manipulation. DSC scans were run immediately after closing the DSC pan. Once the DSC scans were finished, DSC pans were pierced with sterilized tweezers and placed in an oven set at 103 °C for 17 h to allow the complete drying of the spores, as per standard procedures for drying seeds [73]. After drying in the oven, DSC pans were held in a container half filled with silica gel for 15 min to cool down and then weighed again to determine the dry weight of the spore samples.

The presence of phase transitions was determined from cooling and heating thermograms recorded between 25 and −150 °C and between −150 and 90 °C, respectively, while scanning at a rate of 10 °C min^−1^. The onset temperature of melting and freezing transitions was determined from an intersection between the baseline and a line drawn from the steepest portion of the current transition peak. The enthalpy (∆H) of the transition was determined from the area included by the peak and the baseline. Enthalpies of endothermic and exothermic events are expressed on a per g dry weight basis. All analyses were performed using STARe evaluation software (version 16.0, Mettler Toledo). As the water content (WC) progressively increased in spores of *P. formosum* (the spores scanned at diverse water content), the transition size increased, which increased the enthalpy of the main melting event (∆Hm) and moved towards 0 °C. This increase in ∆Hm was attributed to the melting of ice crystals. However, the temperature and ∆H of transitions at very low WC did not change with slight increases in WC and so were attributed to triacylglycerols (TAG) [40]. The slope of the linear relationship between WC and the ∆Hm at the high WC end was used to calculate the ∆H of the water melting transition on a g^−1^ H_2_O basis. The intersection between this line and the horizontal line attributed to the TAG transitions was the exact point below which melting or freezing transition could not be observed [40]. Because spores of the other species were at WC within the low WC region in which ∆Hm were attributed to TAG in *P. formosum*, we assumed that all phase transitions measured in the dry spores were related to TAG. TAG with β’ melting transitions (main melting transition, Figure 1a) in the temperature ranges observed for bryophyte spores are trilinolenin (−34 °C, as per [48]).

### 4.4. Fluorescence and Optical Microscopy 

Chlorophyll and chloroplast presence in bryophyte spores were examined by optical and fluorescence microscopy [74,75]. Spores extracted from the sporangia (often with sporangia residues) were mounted in a drop of glycerin and examined using an epifluorescence microscope (Olympus BX40F-3, Japan). A sample had to contain at least 10 spores to be considered valid. The same microscope was used to obtain optical and fluorescence microscopy images, applying white light from the bottom lamp of the microscope (transmitted light) or light from a 100 W Mercury lamp powered by an Olympus U-RFL-T, Germany (reflected light) after passing through a blue excitation filter set (BP 450–480, DM500 dichromatic beam splitter-dichroic mirror, BA515 barrier filter; Olympus), respectively. Images were captured using an Olympus XC30 digital camera through the software cellSens (Olympus Corporation). Red autofluorescence of chlorophyll permitted the identification of green plastids that could often be observed by optical microscopy (Figure 4). 

## Figures and Tables

**Table 1:** Percentage and rate of germination of spores for the 23 bryophytes species assayed in this work. The final percentage (%) of germination was calculated as the maximum germination reached during the germination tests performed. The rate of germination was determined as the time (in days) by which spores reached 50% of the final germination percentage (t_50_). Significant differences between germination % found in a proportion test (*p* < 0.05) are represented by different letters.

Species Name	Germination (Above: Final %, Below: Rate as t_50_ [Days])	
	Before LN Exposure	After LN Exposure
*Atrichum undulatum* (acc. 1)	25 ^a^ 3.20	2 ^b^ 4.85
*Bryum capillare* (acc. 1)	100 3.10	100 5.60
*B. capillare* (acc. 2)	100 2.60	100 2.60
*B. capillare* (acc. 4)	100 ^a^ 2.3	84 ^b^ 2.3
*B. capillare* (acc. 5)	100 2.55	100 2.6
*B. capillare* (acc. 7)	100 4.8	100 5.2
*Ceratodonpurpureus* (acc. 1)	100 ^a^ 3	71 ^b^ 2.8
*Dicranum scoparium* (acc. 4)	39 ^a^ 4.6	42 ^b^ 6.5
*Funaria hygrometrica* (acc. 1)	100 2.7	100 4
*F. hygrometrica* (acc. 3)	100 2.5	100 3
*F. hygrometrica* (acc. 7)	75 4.7	74 3.45
*F. hygrometrica* (acc. 8)	100 4.9	100 6.75
*Kindbergia praelonga* (acc. 2)	100 ^a^ 6.25	60 ^b^ 7.1
*K. praelonga* (acc. 3)	21 5	0 -
*Leptobryum pyriforme*	100 ^a^ 4.85	56 ^b^ 5.95
*Marchantia polymorpha* (acc. 1)	7 2.4	0 -
*Mnium hornum* (acc. 2)	100 5.61	100 5.95
*Orthodontium lineare* (acc. 3)	79 ^a^ 3.9	73 ^b^ 6.3
*O. lineare* (acc. 3, test 2)	54 10.65	57 6.2
*Polytrichum formosum* (acc. 1)	100 ^a^ 6	76 ^b^ 8.2
*P. formosum* (acc. 1, test 2)	100 5.75	100 5.65
*P. formosum* (acc. 2)	100 5.45	100 5.6
*Tortula muralis* (acc. 2)	100 6.4	100 6.25

**Table 2:** Summary of the calorimetric properties measured for the bryophyte spores tested.

Component	Parameter	*B. capillare*	*O. lineare*	*P. formosum*	*D. scoparium*
Lipid	Melting temperature (°C)	−35.12 ± 0.01	−34.43 ± 0.04	−38.60 ± 4.61	-
	Melting enthalpy (J g^−1^ DW)	10.1 ± 0.4	3.6 ± 3.0	25.8 ± 4.2	0
	Predominant fatty acid ^(a)^	Linolenic	Linolenic	Linolenic	-
	Enthalpy of melt ^(a)^	88	88	88	-
	Lipid content%	11.5	4.1	29.3	0
Water	∆HH_2_O (J g^−1^ H_2_O)	-	-	329	-
	Unfrozen WC (g H_2_O gDW^−1^)	-	-	0.147	-

^(a)^ [48].

**Table 3:** Summary of collection data for the species and accessions of bryophytes harvested for this work. Collection data include location, exposition, coordinates, date of collection and ecology. Additionally, the presence of chloroplasts and chlorophyll in the spore samples is indicated.

Species Name	Accession	Location	Exposition	Coordinates	Collection Date	Ecology *	Chloroplasts/ Chlorophyll ^++^
*Atrichum undulatum* (Hedw.) P. Beauv. ^(a)^	1 ^(a)^	Wakehurst Place, way to Westwood Lake	22 N	51°4′13″N 0°5′32″W	30 June 2021	Terrestrial, terricolous	Y/Y
	2	Wakehurst Place, Mansion	348 N	N 51°3′58″ W 0°5′29″	1 July 2021		-
	3	Ardingly reservoir	305 NW	N 51°3′36″ W 0°5′55″	22 July 2021		-
*Barbula unguiculata* Hedw.	-	Wakehurst Place, water garden	341 N	N 51°3′48′′ W 0°5′24′′	5 July 2021	Aquatic, saxicolous	Y/Y
*Brachytheciastrum velutinum* (Hedw.) Ignatov & Huttunen.	-	Ardingly reservoir	201 S	N 51°3′0.9″ W 0°6′7″	20 July 2021	Epiphytic	Y/Y
*Brachythecium rutabulum* (Hedw.) Schimp.	1	Wakehurst Place, water garden	359 N	N 51°3′49″ W 0°5′25″	5 July 2021	Ubiquitous	Y/Y
	2		310 NW	N 51°3′59″ W 0°5′23″			-
*Bryum argenteum* Hedw.	1	Wakehurst Place, water garden	226 SW	N 51°3′59″ W 0°4′56″	26 July 2021	Terricolous	N/N
*Bryum capillare* Hedw. ^(a), (b)^	1 ^(a)^	Wakehurst Place, MSB greenhouse	161 S	N 51°4′7.9″ W 0°5′25″	30 July 2021	Terricolous	Y/Y
	2 ^(a), (b)^		179 S	N 51°4′7.9″ W 0°5′24″			Y/Y
	3	Wakehurst Place, water garden	79 E	N 51°3′50″ W 0°4′56″	5 July, 2021		Y/Y
	4 ^(a)^	Wakehurst Place, greenhouses	265 O	N 51°4′3.2″ W 0°5′24″	6 July 2021		Y/Y
	5 ^(a)^		245 SW	N 51°4′4.4″ W 0°5′24″			N/N
	6		232 SW	N 51°4′3.8″ W 0°5′25″			-
	7 ^(a)^	Wakehurst Place, Mansion	10 N	N 51°3′59″ W 0°4′56″	26 July 2021		-
*Ceratodon purpureus* (Hedw.) Brid. ^(a)^	1 ^(a)^	Wakehurst Place, Mansion	261 W	N 51°4′1.69″ W 0°5′25″	1 July 2021	Terricolous	Y/Y
	2		56 NE	N 51°3′56″ W 0°5′25″			-
	3	Wakehurst Place, greenhouses	255 W	N 51°4′4.2″W 0°5′26″	6 July 2021		-
*Dicranella heteromalla* (Hedw.) Schimp.	1	Wakehurst Place, water garden	322 NW	N 51°3′49″ W 0°5′35″	5 July 2021	Terrestrial	-
	2	Wakehurst Place, greenhouses	265 W	N 51°4′2.3″ W 0°5′24″	6 July 2021		Y/Y
	3	Ardingly reservoir	305 NW	N 51°3′36″ W 0°5′55″	22 July 2021		Y/Y
	4	Wakehurst Place, Himalayan Glade	21 N	N 51°3′52″ W 0°5′41″	26 July 2021		-
*Dicranum scoparium* Hedw. ^(a), (b)^	1	Wakehurst Place, MSB greenhouse	192 S	N 51°4′7.3″ W 0°5′24″	30 June 2021	Ubiquitous	-
	2	Chidingly Wood	216 SW	N 51°3′59″ W 0°4′56″	8 July 2021		-
	3		216 SW	N 51°3′59″ W 0°4′56″			-
	4 ^(a), (b)^	Wakehurst Place, water garden	303 NW	N 51°3′51″ W 0°5′21″	26 July 2021		Y/Y
*Fissidens taxifolius* Hedw.	-	Ardingly reservoir	274 W	N 51°3′9.1″ W 0°7′10″	22 July 2021	Terricolous	Y/Y
*Funaria hygrometrica* Hedw. ^(a)^	1 ^(a)^	Wakehurst Place, MSB greenhouse	281 W	N 51°4′8.18″ W 0°5′24.9″	30 July 2021	Terricolous	N/N
	2	Wakehurst Place, Wall Garden	207 S	N 51°3′59.9″ W 0°4′57″	1 July 2021		-
	3 ^(a)^	Wakehurst Place, Mansion	348 N	N 51°3′58″ W 0°5′29″	1 July 2021		N/N
	4	Wakehurst Place, greenhouses	146 SE	N 51°3′48″ W 0°5′28″	5 July 2021		-
	5		182 S	N 51°4′2.5″ W 0°5′25″	6 July 2021		-
	6		204 SW	N 51°4′2.3″ W 0°5′24″			-
	7 ^(a)^		245 SW	N 51°4′4.4″ W 0°5′24″			N/N
	8 ^(a)^	Ardingly reservoir	134 SE	N 51°3′9.1″ W 0°7′10″	20 July 2021		Y/Y
*Hypnum cupressiforme* Hedw.	1	Wakehurst Place, way to Westwood Lake	27 NE	N 51°4′9.5″ W 0°5′30″	30 June 2021	Epiphytic and epiphyllous	-
	2		113 SE	N 51°4′0.17″ W 0°5′1.43″			-
	3		205 SW	N 51°4′2.7″ W 0°6′0.26″			Y/Y
	4	Wakehurst Place, Himalayan Glade	214 SW	N 51°3′47″ W 0°5′41.97″	30 June 2021		-
	5	Wakehurst Place, Mansion	154 SE	N 51°4′1.65″ W 0°5′25″	1 July 2021		Y/Y
	6	Wakehurst Place, water garden	219 SW	N 51°3′49″ W 0°5′21″	5 July 2021		-
	7		259 W	N 51°4′20″ W 0°5′59″			-
	8		358 N	N 51°3′48″ W 0°5′24″			Y/Y
*Isothecium myosuroides* Brid.	-	Ardingly reservoir	250 W	N 51°3′39″ W 0°7′10″	22 July 2021	Epiphytic on bark	Y/Y
*Kindbergia praelonga* (Hedw.) Ochyra ^(a)^	1	Wakehurst Place, Westwood Lake	357 N	N 51°3′13″ W 0°7′8.3″	30 July 2021	Terricolous	Y/Y
	2 ^(a)^	Wakehurst Place, water garden	356 N	N 51°3′55″ W 0°4′56″	5 July 2021		-
	3 ^(a)^	Wakehurst Place, Westwood Lake	135 SE	N 51°4′48″ W 0°6′2.48″	19 July 2021		Y/Y
*Leptobryum pyriforme* (Hedw.) Wilson ^(a)^	- ^(a)^	Wakehurst Place, greenhouses	182 S	N 51°4′2.5″ W 0°5′25″	6 July 2021	Terricolous	-
*Marchantia polymorpha* L. ^(a)^	1 ^(a)^	Wakehurst Place, MSB greenhouse	216 SW	N 51°4′7.8″ W 0°5′24″	30 July 2021	Terricolous, sometimes submerged	-
	2	Wakehurst Place, greenhouses	182 S	N 51°4′2.5″ W 0°5′25″	6 July 2021		-
*Mnium hornum* Hedw. ^(a)^	1	Wakehurst Place, water garden	55 E	N 51°3′59″ W 0°4′57″	5 July 2021	Terricolous	-
	2 ^(a)^	Wakehurst Place, MSB greenhouse	192 S	N 51°4′7.3″ W 0°5′24.9″	16 July 2021 ^(a)^		Y/Y
*Orthodontium lineare* Schwägr. ^(a), (b)^	1	Chidingly Wood	53 NE	N 51°4′23″ W 0°4′39″	8 July 2021	Saxicolous	-
	2		79 E	N 51°3′59″ W 0°4′56″			Y/Y
	3 ^(a), (b)^	Wakehurst Place, Himalayan Glade	170 S	N 51°3′51″ W 0°5′42″	26 July 2021		N/N
*Orthotrichum affine* Schrader ex Bridel	1	Wakehurst Place, way to Mansion	33 NE	N 51°3′13″ W 0°5′41″	30 June 2021	Epiphytic on bark	-
	2		240 S	N 51°3′59″ W 0°4′57″	1 July 2021		Y/Y
	3	Wakehurst Place, Himalayan Glade	33 NE	N 51°3′13″ W 0°5′41″	30 July 2021		Y/Y
*Oxyrrhynchium hians* (Hedw.) Loeske	1	Wakehurst Place, water garden	359 N	N 51°3′59″ W 0°4′57″	5 July 2021	Terrestrial	-
	2	Ardingly reservoir	140 SE	N 51°3′38″ W 0°5′54″	22 July 2021		-
*Plagiomnium undulatum* (Hedw.) T.J.Kop.	-	Wakehurst Place, water garden	297 NW	N 51°3′48″ W 0°4′57″	5 July 2021	Saxicolous	-
*Polytrichum formosum* (Hedw.) G.L. Sm. ^(a), (b)^	1 ^(a), (b)^	Wakehurst Place, Westwood Lake	328 NW	N 51°3′47″ W 0°5′57″	30 July 2021	Terricolous	Y/Y
	2 ^(a), (b)^	Wakehurst Place, winter garden	11 N	N 51°3′59″ W 0°4′57″	1 July 2021	Terricolous	Y/Y
*Tortula muralis* Hedw. ^(a)^	1	Wakehurst Place, Mansion	360 N	N 51°3′58″ W 0°5′22″	30 June 2021	Saxicolous	Y/Y
	2 ^(a)^		72 E	N 51°3′59″ W 0°4′57″	1 July 2021		N/Y
*Ulota bruchii* Hornsch. ex Brid.	1	Wakehurst Place, Himalayan Glade	160 S	N 51°3′17″ W 0°7′2.14″	30 July 2021	Epiphytic	-
	2	Wakehurst Place, Westwood Lake	358 N	N 51°4′26.7″ W 0°5′12.6″	19 July 2021		Y/Y
*Ulota crispa* (Hedw.) Brid.	-	Wakehurst Place, Japan garden	195 N	N 51°3′45″ W 0°4′59″	5 July 2021	Epiphytic	Y/Y

^(a)^ indicates species used for germination tests, ^(b)^ indicates species used for DSC experiments. ^++^ chloroplast presence (Y) observed by light microscopy and chlorophyll presence (Y) detected by red autofluorescence in a fluorescent microscope. N indicates chloroplast or chlorophyll absence. * the terminology used for the descriptions of “Ecology” is adopted from [49] as follows: “Epiphytic”, when a species grows on trees, at diverse heights and/or preferring some positions around the tree itself, where growing on leaves the term changes to epiphyllous; “Saxicolous”, when a species grows on rocks or rocky substrata; “Terricolous”, said of a species growing on soil; “Terrestrial”, when a species grows both on soil and/or rocks and/or plants; “Aquatic”, when a species lives submerged or near streams, rivers or aquatic environments; “Ubiquitous”, when a species grows in different types of habitats and substrata, ranging from streams to branches of trees, from rocks to soil.

## Data Availability

The data presented in this study are available on request from the corresponding author. The data are not publicly available due to privacy.
